# Association between intravenous magnesium sulfate and mortality in patients with sepsis-associated liver injury: a retrospective cohort study

**DOI:** 10.3389/fmed.2025.1679032

**Published:** 2026-01-13

**Authors:** Chen Ouyang, Yuxi Liu, Xin Li, Pengpeng Liu, Quanyan Liu

**Affiliations:** 1 Department of Hepatobiliary Surgery, Tianjin Medical University General Hospital, Tianjin, China; 2 Department of Critical Care and Immunology, Clinical Immunology Center, Tianjin Medical University General Hospital, Tianjin, China

**Keywords:** magnesium, MIMIC-IV, mortality, sepsis-associated liver injury, sulfate

## Abstract

**Background:**

Sepsis-associated liver injury (SALI) is a significant risk factor for mortality in patients with sepsis. Magnesium, as an essential electrolyte, has a correlation with adverse outcomes in critical illness when deficient, yet the therapeutic efficacy of magnesium sulfate in SALI remains undetermined. This study was designed to evaluate the association between magnesium sulfate therapy and prognosis in SALI patients.

**Method:**

This retrospective cohort study utilized data from the Medical Information Mart for Intensive Care IV (MIMIC-IV) database, with the primary endpoint being 30-day all-cause mortality.

Propensity score matching (PSM) achieved covariate balance, Kaplan-Meier survival curves and Cox regression were employed to analyze the magnesium sulfate-mortality relationship in SALI patients. The study results were externally validated using the eICU 2.0 database.

**Result:**

The present study was conducted on 648 SALI patients. After PSM, the 30-day all-cause mortality rate was significantly reduced in the magnesium sulfate group versus the non-magnesium sulfate group (30.4% vs. 44.6%, *P* = 0.002). Kaplan-Meier survival analysis demonstrated superior 30-day cumulative survival rates in the magnesium sulfate group after PSM (*P* < 0.001). Both multivariable Cox regression (HR, 0.62, 95% CI, 0.47–0.82, *P* < 0.001) and inverse probability weighting (IPW) analysis (HR, 0.69, 95% CI, 0.53–0.89, *P* = 0.005) indicated that magnesium sulfate treatment was an independent protective factor for 30-day all-cause mortality risk.

**Conclusion:**

The use of magnesium sulfate is associated with a reduction in all-cause mortality among SALI patients. Future research should consider individual patient variations to explore its true effectiveness.

## 1 Introduction

Sepsis represents a clinical syndrome of dysregulated infection response, manifesting as systemic inflammation, multi-organ dysfunction, and tissue injury (1). Epidemiological studies report an annual sepsis incidence of about 189 cases per 100,000 adults, with mortality reaching 26.7%, and 24.4% of cases occurring in intensive care units (ICUs) (2). As a central metabolic and immunological organ, the liver plays a critical role in sepsis by facilitating bacterial clearance and synthesizing acute-phase proteins and cytokines, rendering it one of the most frequently affected organs in this pathological state (3). Liver injury can occur at any stage of sepsis and is an independent risk factor for multiple organ dysfunction and sepsis-related mortality (4). The pathogenesis of sepsis-associated liver injury (SALI) involves an intricate interaction of inflammatory cytokines, oxidative stress, and mitochondrial dysfunction. Excessive inflammatory responses lead to hepatocyte damage (5), exacerbating the patient’s condition by impairing protein synthesis, disrupting glucose metabolism, and causing the accumulation of toxic substances (6). Studies indicate that the mortality rate for SALI patients ranges from 54% to 68% (4). Minimizing liver injury and promoting liver function recovery are critical for reducing mortality in sepsis patients.

Magnesium is the second most prevalent intracellular electrolyte and functions as a cofactor for a wide array of enzymes, playing an essential role in preserving cellular structure, regulating metabolism, and maintaining energy homeostasis (7). Magnesium can reduce the production of inflammatory cytokines in monocytes via the Toll-like receptor pathway (8), block N-terminal gasdermin-D-induced pyroptosis (9), and inhibit the NF-κB signaling pathway. This effectively diminishes the production of pro-inflammatory cytokines such as TNF-α, thereby mitigating tissue damage caused by uncontrolled inflammatory responses (10). Magnesium supplementation downregulates neutrophil respiratory burst, alleviates oxidative stress (11), and significantly enhances lactate clearance (12). This improves tissue energy metabolism and hypoxic conditions while reducing hepatic metabolic burden. Hypomagnesemia is independently associated with septic coagulopathy. Maintaining magnesium homeostasis may protect the liver by improving microcirculation and reducing microthrombus formation (13). These mechanisms suggest magnesium supplementation may offer therapeutic benefits for SALI.

Currently, effective treatment options for SALI remain limited, and there is insufficient evidence-based data on whether magnesium sulfate therapy can improve the survival rate of SALI patients. There is an urgent need to examine more effective interventions aimed at ameliorating the prognosis of patients with SALI. Therefore, this study based on the Medical Intensive Care Unit Information Database (MIMIC-IV), aims to systematically assess the correlation between magnesium sulfate therapy and clinical mortality in SALI patients.

## 2 Materials and methods

### 2.1 Data source

This is a retrospective observational cohort study based on the MIMIC IV 3.1 database (14). This public clinical database includes all intensive care cases from Beth Israel Deaconess Medical Center in the United States between 2008 and 2022. The researchers have completed the training program assessment for collaborating institutions (certification number: 14363479) and obtained authorization to use the database. Since the MIMIC-IV database uses anonymized data, patient informed consent is not required. Data extraction was conducted via Structured Query Language (SQL), utilizing script code obtained from https://github.com/MIT-LCP/mimic-code/. This study protocol was approved by the Ethics Committee of Tianjin Medical University General Hospital.

### 2.2 Study patients

Inclusion criteria: (1) Patient meets diagnostic criteria for sepsis 3.0 (15); (2) Patients meeting the SALI diagnostic criteria: INR > 1.5 and total bilirubin > 2 mg/dL (34.2 μmol/L) (16); (3) SALI diagnosed within 24 h of ICU admission. Exclusion criteria: (1) readmission to the hospital or ICU; (2) age < 18 years; (3) ICU stay < 24 h; (4) combined with other liver diseases: ICD-9 codes 570 (acute hepatic failure and necrosis), 571 (chronic liver disease and cirrhosis), 572 (liver abscess and sequelae), 573 (other liver disorders); ICD-10 codes K70 (alcoholic liver disease), K71 (toxic liver disease), K72 (hepatic failure), K73 (chronic hepatitis), K74 (liver fibrosis and cirrhosis), K75 (other inflammatory liver diseases), K76 (other liver diseases), K77 (liver disorders in other diseases classified elsewhere).

### 2.3 Variables

At the time of patients’ initial admission to the intensive care unit (ICU), the following variables were collected: heart rate (HR), Respiratory Rate (RR), international normalized ratio (INR), total bilirubin (TB), mean blood pressure (MBP), Temperature (T), oxygen saturation (SpO_2_), platelets, white blood cell (WBC), hemoglobin, alanine aminotransferase (ALT), aspartate aminotransferase (AST), lymphocytes, monocytes, neutrophils, alkaline phosphatase (ALP), sequential organ failure assessment (SOFA), Simplified Acute Physiology Score II (SAPS II), charlson comorbidity index (CCI); Demographics: age, race, gender; Comorbidity: cerebrovascular disease (CVD), hypertension, coronary artery disease (CAD), diabetes, acute kidney injury (AKI); Critical treatments on the first day: vasopressor used, renal replacement therapy (RRT) used, Mechanical ventilation used. We excluded cases with more than 25% missing variables and used Predictive Mean Matching (PMM) for multiple imputation in cases with less than 25% missing variables, as shown in Supplementary Figure 1A.

### 2.4 Exposure and outcomes

Exposure was defined as the administration of intravenous magnesium sulfate during ICU hospitalization among patients with SALI. Data regarding intravenous magnesium sulfate administration were extracted from the “inputevents” database. The all-cause mortality rate within 30 days is the primary outcome. The 90-day all-cause mortality, ICU mortality, in-hospital mortality, and lengths of stay in both the hospital and ICU are secondary outcomes.

### 2.5 Propensity score matching

Propensity score matching (PSM) was applied to correct for variables between the magnesium sulfate and non-magnesium sulfate groups (17), including age, gender, race, ALT, AST, hemoglobin, platelets, WBC, AKI, CCI, hypertension, and CAD. Propensity scores were generated for each participant via a logistic regression model, with the two cohorts matched in a 1:1 ratio. The covariate balance between the matched groups was evaluated using the standardized mean difference (SMD), where SMD < 0.10 was considered to indicate adequate balance.(18), as shown in Supplementary Figures 1B, C.

### 2.6 External validation

The population data were extracted from the eICU 2.0 database. Patients with SALI were defined and selected through ICD-9 codes. Demographic data, laboratory test results, treatment plans, and clinical outcomes were extracted using SQL. The inverse probability weighting (IPW) analysis and multivariate Cox regression model were used to evaluate the correlation between magnesium sulfate treatment and all-cause mortality during hospitalization, ICU stay, and 30 days.

### 2.7 Statistical analysis

The distribution of continuous variables was ass with the Shapiro-Wilk test. Non-normally distributed variables were reported as median (interquartile range, IQR), with analysis conducted using the Mann-Whitney U test. Categorical variables were presented as percentages and compared using Fisher’s exact test.

A multivariate Cox proportional hazards model was employed to investigate the association between magnesium sulfate administration and mortality, with results expressed as hazard ratios (HRs) accompanied by 95% confidence intervals (CIs). Survival outcomes were analyzed using Kaplan-Meier estimators, and group differences were assessed via the log-rank test. Furthermore, restricted cubic spline (RCS) regression was applied to evaluate the non-linear relationships between INR or TB levels and 30-day mortality risk. Subgroup analyses were conducted based on demographic variables (age, sex, race), clinical parameters (INR, TB, SOFA, SAPS II, and CCI), comorbid conditions (hypertension, CAD, diabetes, AKI, RRT, vasopressor usage, and mechanical ventilation), as well as magnesium sulfate dosing characteristics (average daily dose and duration of treatment). Statistical analyses were performed using a two-tailed approach, with statistical significance defined as *P* < 0.05. The study data were obtained from the MIMIC-IV database through extraction using Structured Query Language (SQL) and Navicat Premium (version 17.0)., and all subsequent statistical analyses were performed using R software (version 4.4.2).

## 3 Results

### 3.1 Baseline characteristics of patients

**FIGURE 1:**
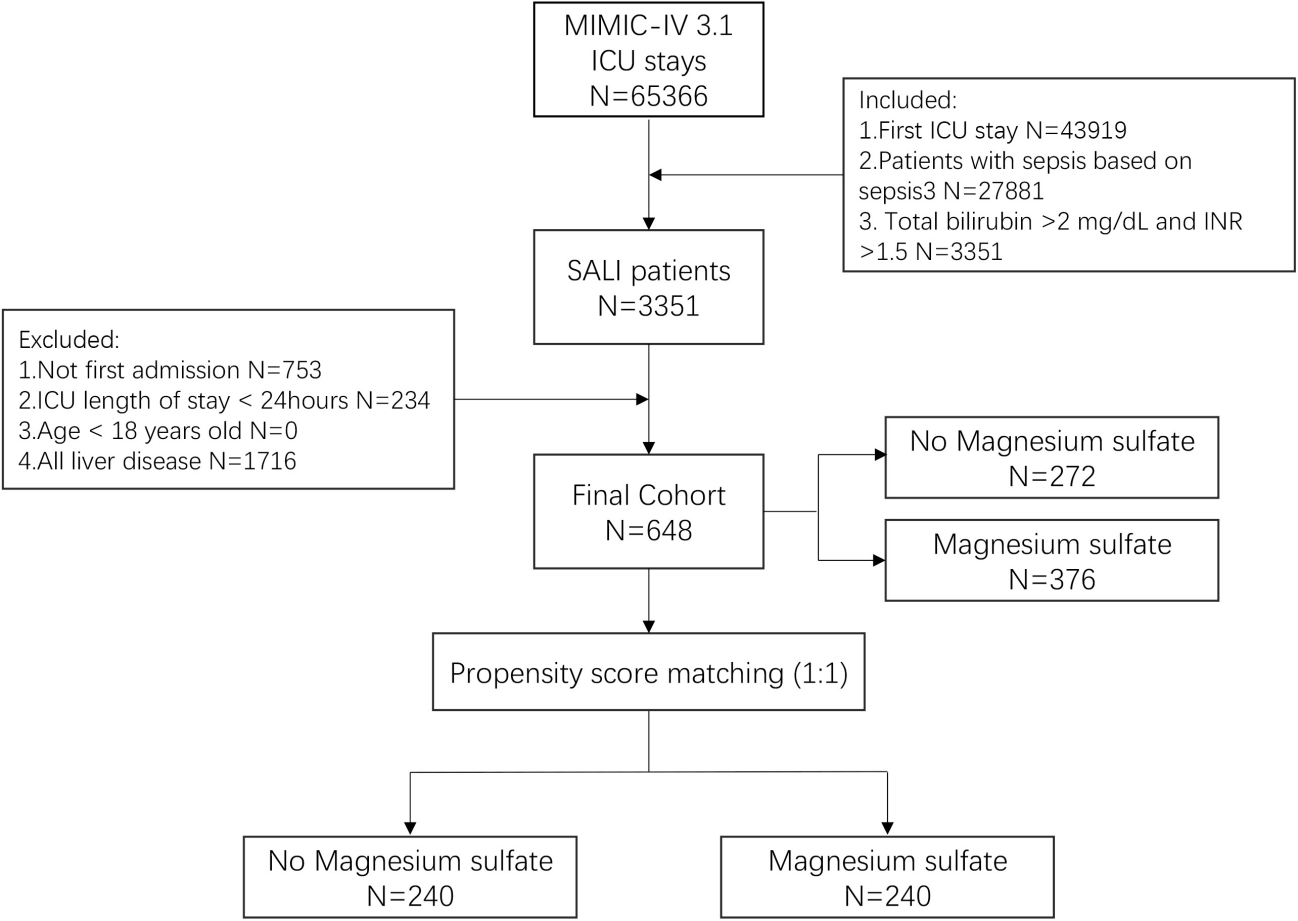
Flow chart of patient selection. MIMIC-IV, Medical Information Mart in Intensive Care-IV; ICU, intensive care unit; INR, international normalized ratio; SALI, sepsis-associated liver injury.

This study involved 648 patients with SALI, among whom 376 (58%) were treated with magnesium sulfate during hospitalization, while 272 (42%) were not. The screening process is shown in Figure 1. Patients receiving magnesium sulfate had a median age of 67.6 years, with 57.2% males and 63% white individuals, showing median values of 2.0 for INR and 3.6 mg/dL for TB. In comparison, the control group (non-magnesium sulfate) demonstrated a median age of 69.8 years, with 65.1% males and 57.4% white individuals, along with median INR of 2.2 and TB of 3.9 mg/dL, as shown in Table 1. Notably, baseline characteristic analysis revealed that patients in the magnesium sulfate treatment group presented with more severe baseline conditions, manifested by higher SOFA scores (4.0 vs. 3.0, *p* = 0.035), more frequent vasopressor use (60.1% vs. 39.3%, *p* < 0.001) and mechanical ventilation (16.8% vs. 10.7%, *p* = 0.030). The paradoxical lower mortality rate in this group supports that magnesium sulfate may be associated with survival benefits, rather than being attributable to selection bias. The matched cohort consisted of 480 patients evenly distributed between the two groups, with 240 individuals in each. Following PSM, baseline characteristics were comparable across both groups.

**TABLE 1:** The baseline characteristics.

	Before PSM			After PSM		
Variables	No magnesium	Magnesium	p	No magnesium	Magnesium	*p*
Patients number	272	376		240	240	
**Demographics**						
Age (years)	69.8 (59.3, 81.5)	67.6 (53.1, 79.0)	0.071	69.3 (58.5, 81.4)	69.2 (56.7, 79.5)	0.514
**Race**						
Black	27 (9.9)	32 (8.5)	0.345	21 (8.8)	20 (8.3)	0.702
Unknow/Other	89 (32.7)	107 (28.5)		77 (32.1)	69 (28.7)	
White	156 (57.4)	237 (63.0)		142 (59.2)	151 (62.9)	
Gender, male	177 (65.1)	215 (57.2)	0.051	153 (63.7)	138 (57.5)	0.191
Laboratory variables INR	2.2 (1.8, 3.2)	2.0 (1.8, 2.7)	0.020	2.2 (1.8, 3.2)	2.1 (1.8, 2.7)	0.061
Bilirubin (mg/dL)	3.9 (2.7, 6.3)	3.6 (2.6, 5.3)	0.098	3.6 (2.6, 5.3)	3.6 (2.6, 5.4)	0.761
Heart rate (bpm)	91.1 (80.7, 108.1)	94.4 (81.6, 106.2)	0.381	91.7 (80.7, 109.5)	95.0 (82.0, 105.4)	0.668
MBP (mmHg)	74.6 (70.3, 80.4)	74.0 (68.7, 80.9)	0.190	74.7 (70.2, 80.4)	74.0 (69.6, 80.8)	0.354
RR (breaths/min)	21.6 (18.0, 24.6)	21.1 (18.3, 24.9)	0.917	21.8 (18.1, 24.8)	21.1 (18.6, 25.3)	0.941
Temperature (°C)	36.8 (36.5, 37.2)	36.8 (36.5, 37.2)	0.839	36.8 (36.5, 37.2)	36.8 (36.5, 37.1)	0.823
SpO2 (%)	96.6 (95.0, 98.0)	96.9 (95.3, 98.3)	0.134	96.5 (95.0, 98.0)	96.8 (95.2, 98.1)	0.298
WBC (10^∧^9/L)	13.6 (8.1, 20.7)	13.4 (7.8, 19.8)	0.535	13.8 (7.9, 20.5)	13.4 (8.3, 20.2)	0.824
Platelets (10^∧^9/L)	140.5 (87.8, 206.3)	143.5 (88.8, 208.3)	0.662	143.5 (90.8, 207.0)	141.0 (83.0, 215.5)	0.856
Hemoglobin (g/dL)	10.4 (8.5, 12.3)	10.6 (8.6, 12.4)	0.615	10.7 (8.8, 12.5)	10.7 (8.6, 12.1)	0.509
Lymphocytes (%)	6.9 (4.0, 11.7)	6.4 (3.0, 10.0)	0.051	6.7 (4.0, 11.5)	6.3 (3.0, 10.2)	0.099
Monocytes (%)	4.1 (2.0, 7.0)	4.0 (2.2, 6.6)	0.710	4.1 (2.0, 7.0)	4.0 (2.3, 7.0)	0.910
Neutrophils (%)	92.0 (87.0, 97.0)	92.0 (88.0, 97.0)	0.520	92.0 (87.0, 96.0)	92.0 (88.0, 98.0)	0.315
ALT (U/L)	227.0 (82.5, 774.0)	164.5 (70.8, 485.3)	0.026	202.5 (77.0, 718.3)	174.0 (73.0, 589.3)	0.483
AST (U/L)	189.0 (72.0, 735.5)	149.0 (63.8, 471.3)	0.076	184.5 (66.8, 680.3)	162.5 (67.8, 563.8)	0.606
ALP (U/L)	168.5 (91.0, 304.5)	116.0 (67.0, 195.0)	<0.001	159.5 (89.8, 279.5)	125.0 (69.0, 206.3)	0.001
SAPS II score	52.0 (41.0, 66.0)	50.5 (39.0, 63.0)	0.056	50.5 (40.0, 65.0)	51.0 (40.0, 64.0)	0.748
SOFA score	3.0 (0.0, 6.0)	4.0 (1.0, 6.0)	0.035	3.0 (0.0, 6.0)	4.0 (1.0, 7.0)	0.001
CCI score	1.0 (0.0, 4.0)	0.0 (0.0, 2.0)	<0.001	0.0 (0.0, 3.0)	0.0 (0.0, 3.0)	0.578
**Comorbidity**						
Hypertension	160 (58.8)	211 (56.1)	0.520	139 (57.9)	139 (57.9)	1.000
CAD	119 (43.8)	137 (36.4)	0.062	99 (41.2)	104 (43.3)	0.712
Cerebrovascular	31 (11.4)	23 (6.1)	0.021	26 (10.8)	16 (6.7)	0.145
Diabetes	95 (34.9)	98 (26.1)	0.019	78 (32.5)	63 (26.2)	0.161
AKI	200 (73.5)	242 (64.4)	0.017	173 (72.1)	165 (68.8)	0.484
**Critical treatments**						
Vasopressor	107 (39.3)	226 (60.1)	<0.001	95 (39.6)	145 (60.4)	<0.001
RRT	21 (7.7)	36 (9.6)	0.483	18 (7.5)	30 (12.5)	0.093
Mechanical ventilation	29 (10.7)	63 (16.8)	0.030	27 (11.2)	44 (18.3)	0.039
Diuretic	140 (31.3)	82 (40.8)	0.020	83 (34.6)	81 (33.8)	0.923

INR, international normalized ratio; MBP, mean blood pressure; RR, Respiratory Rate; SpO2, oxygen saturation; WBC, white blood cell; ALT, alanine aminotransferase; AST, aspartate aminotransferase; ALP, alkaline phosphatase; SAPS II, Simplified Acute Physiology Score II; SOFA, sequential organ failure assessment; CCI, charlson comorbidity index; CAD, coronary artery disease; CVD, cerebrovascular disease; AKI, acute kidney injury; RRT, renal replacement therapy. *P*-value < 0.05 is considered statistically significant.

### 3.2 outcomes

Before PSM, the median of hospital stay for patients in the magnesium sulfate group was 11 days, the median of ICU stay was 4.7 days, the in-hospital mortality rate was 33.2%, the ICU mortality rate was 27.7%, the 30-day mortality rate was 32.7%, and the 90-day mortality rate was 41.8%. For patients in the non-magnesium sulfate group, the median of hospital stay was 7 days, the median of ICU stay was 2.6 days, the in-hospital mortality rate was 44.5%, the ICU mortality rate was 37.1%, the 30-day mortality rate was 48.9%, and the 90-day mortality rate was 56.3%. After receiving magnesium sulfate therapy, SALI patients had longer hospital and ICU stays (all *P* < 0.001). However, their in-hospital (*P* = 0.005), ICU (*P* = 0.013), 30-day (*P* < 0.001), and 90-day (*P* < 0.001) mortality rates were significantly reduced.

Following PSM, the median hospital stay for patients in the magnesium sulfate group was 11 days, the median ICU stay was 4.2 days, the in-hospital mortality rate was 32.1%, the ICU mortality rate was 27.1%, the 30-day mortality rate was 30.4%, and the 90-day mortality rate was 42.1%. In the non-magnesium sulfate group, the median of hospital stay was 7 days, the median of ICU stay was 2.6 days, the in-hospital mortality rate was 41.7%, and the ICU mortality rate was 35.4%, the 30-day mortality rate was 44.6%, and the 90-day mortality rate was 52.9%. Within the study cohort, magnesium sulfate administration was significantly associated with prolonged hospitalization and ICU stays (all *P* < 0.001), while demonstrating reduced in-hospital mortality (*P* = 0.037), 30-day (*P* = 0.002) and 90-day mortality (*P* = 0.022). As demonstrated in Table 2.

**TABLE 2:** Clinical endpoints of patients.

	Before PSM			After PSM		
Variables	No magnesium	Magnesium	*p*-value	No magnesium	Magnesium	*P*-value
Patients number	272	376		240	240	
Length of hospital stay	7.0 (3.0, 14.0)	11.0 (6.0, 19.0)	<0.001	7.0 (2.8, 14.0)	11.0 (6.0, 19.0)	<0.001
Length of ICU stay	2.6 (1.7, 4.9)	4.7 (2.5, 10.6)	<0.001	2.6 (1.7, 4.9)	4.2 (2.3, 9.9)	<0.001
In-hospital mortality	121.0 (44.5%)	125.0 (33.2%)	0.005	100 (41.7%)	77 (32.1%)	0.037
ICU mortality	101.0 (37.1%)	104.0 (27.7%)	0.013	85 (35.4%)	65 (27.1%)	0.061
30-day mortality	133.0 (48.9%)	123.0 (32.7%)	<0.001	107 (44.6%)	73 (30.4%)	0.002
90-day mortality	153.0 (56.3%)	157.0 (41.8%)	<0.001	127 (52.9%)	101 (42.1%)	0.022

PSM, propensity score matching; ICU, intensive care unit. *P*-value < 0.05 is considered statistically significant.

### 3.3 Survival analysis

**FIGURE 2:**
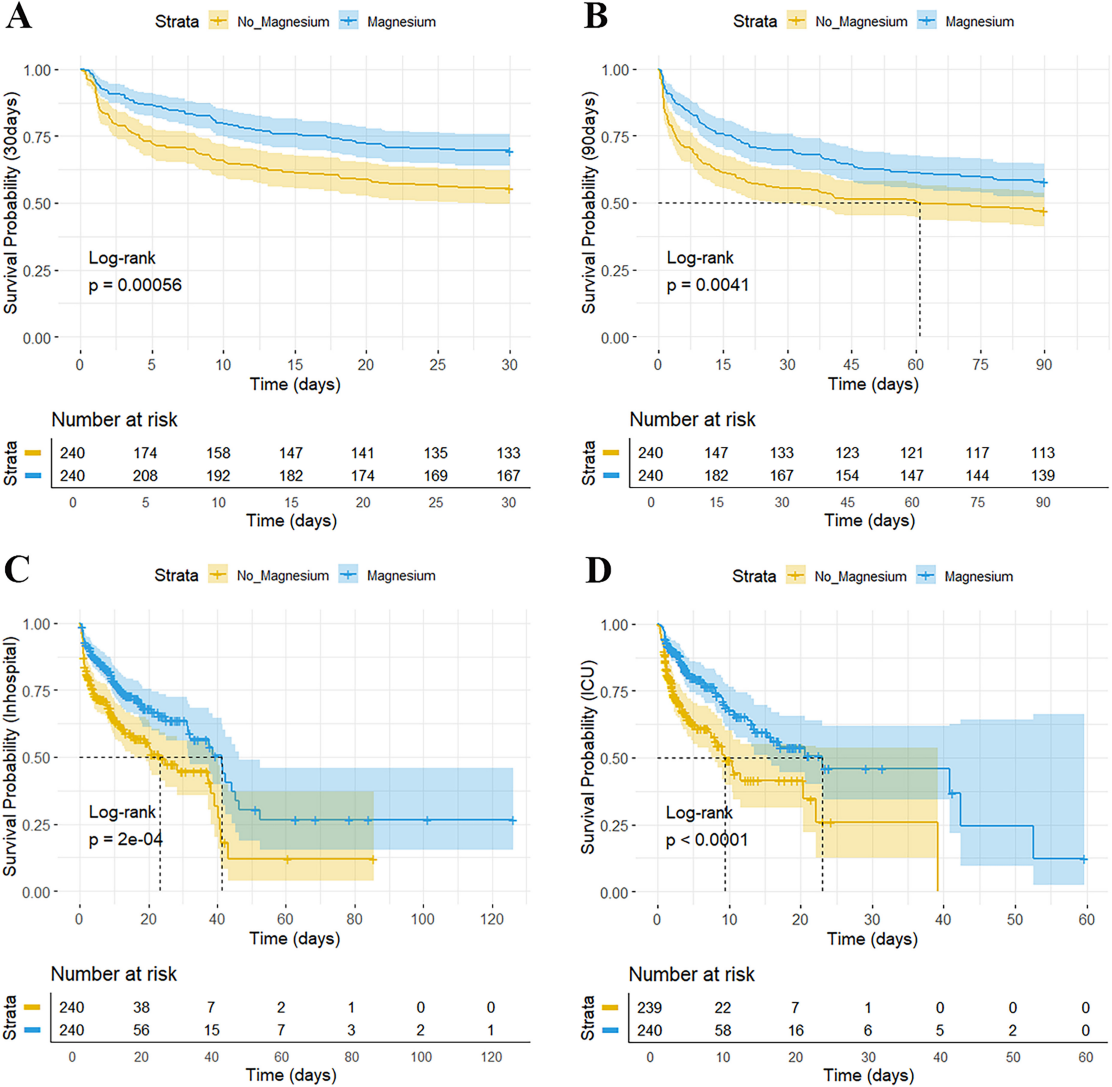
Kaplan-Meier survival analysis after PSM. Kaplan–Meier curves (log-rank test) show 30-day survival probability **(A)**, 90-day survival probability **(B)**, in-hospital survival probability **(C)**, and ICU survival probability **(D)**, grouped by Magnesium sulfate used. The X-axis denotes the time (days), and the Y-axis denotes the cumulative survival probability. ICU, intensive care unit; PSM, propensity score matching. *P*-value < 0.05 is considered statistically significant.

The Kaplan-Meier survival curve shows the cumulative survival rates of the two groups of patients over time. Before PSM, the 30-day and 90-day survival rates, the ICU and hospitalization survival rates of the magnesium sulfate group were significantly higher than those of the non-magnesium sulfate group (all *P* < 0.001), as shown in Supplementary Figure 2. After PSM, the magnesium sulfate group still demonstrated significantly superior 30-day survival rate (*P* = 0.00056), 90-day survival rate (*P* = 0.0041), ICU survival rate (*P* < 0.0001), and in-hospital survival rate (*P* < 0.0001), as shown in Figure 2.

Multivariable Cox regression identified magnesium sulfate treatment as an independent protective factor against mortality in SALI patients, significantly reducing 30-day mortality (HR = 0.62, 95%CI 0.47–0.82, *P* < 0.001), 90-day mortality (HR = 0.69, 95%CI 0.53–0.88, *P* = 0.003), in-hospital mortality (HR = 0.55, 95%CI 0.41–0.74, *P* < 0.001), and ICU mortality (HR = 0.43, 95%CI 0.31–0.60, *P* < 0.001). IPW analysis showed a 30-day mortality rate (HR = 0.69, 95% CI 0.53–0.89, *P* = 0.005), 90-day mortality rate (HR = 0.75, 95% CI 0.59–0.95, *P* = 0.016), in-hospital (HR = 0.61, 95% CI 0.47–0.80, *P* < 0.001) and intensive care unit (ICU) mortality (HR = 0.52, 95% CI 0.39–0.70, *P* < 0.001) mortality rates were significantly reduced, further confirming the robustness of the study results, as shown in Table 3. To examine the relationship between magnesium sulfate dosage and clinical outcomes in patients with SALI, we stratified the PSM-matched cohort into high-dose (*n* = 143, > 12 g/day) and low-dose (*n* = 97, ≤ 12 g/day) groups based on daily average magnesium sulfate administration. Kaplan-Meier survival analysis indicated no statistically significant differences in clinical outcomes across dosage groups, as shown in Supplementary Figure 3.

**TABLE 3:** Correlation between magnesium sulfate and mortality. Non-magnesium sulfate is the reference.

Mortality	MODEL	HR (%95 cl)	*P*-value
30-day mortality	Unadjusted	0.57 (0.45–0.73)	<0.001
	PSM	0.60 (0.44–0.80)	<0.001
	Multivariate adjusted	0.62 (0.47–0.82)	<0.001
	IPW	0.69 (0.53–0.89)	0.005
90-day mortality	Unadjusted	0.62 (0.50–0.78)	<0.001
	PSM	0.68 (0.53–0.89)	0.004
	Multivariate adjusted	0.69 (0.53–0.88)	0.003
	IPW	0.75 (0.59–0.95)	0.016
In hospital mortality	Unadjusted	0.54 (0.42–0.70)	<0.001
	PSM	0.57 (0.42–0.77)	<0.001
	Multivariate adjusted	0.55 (0.41–0.74)	<0.001
	IPW	0.61 (0.47–0.80)	<0.001
ICU mortality	Unadjusted	0.45 (0.34–0.59)	<0.001
	PSM	0.49 (0.35–0.68)	<0.001
	Multivariate adjusted	0.43 (0.31–0.60)	<0.001
	IPW	0.52 (0.39–0.70)	<0.001

PSM, propensity score matching; IPW, inverse probability weighting; ICU, intensive care unit; HR, hazard ratio; CI, confidence interval. *P*-value < 0.05 is considered statistically significant.

Furthermore, both INR and total bilirubin levels demonstrated significant associations with mortality in SALI patients (all *P* < 0.001). RCS analysis indicated a linear relationship between INR and 30-day mortality, while total bilirubin showed a non-linear “U-shaped” association, as shown in Supplementary Figure 4.

### 3.4 Subgroup analysis

**FIGURE 3:**
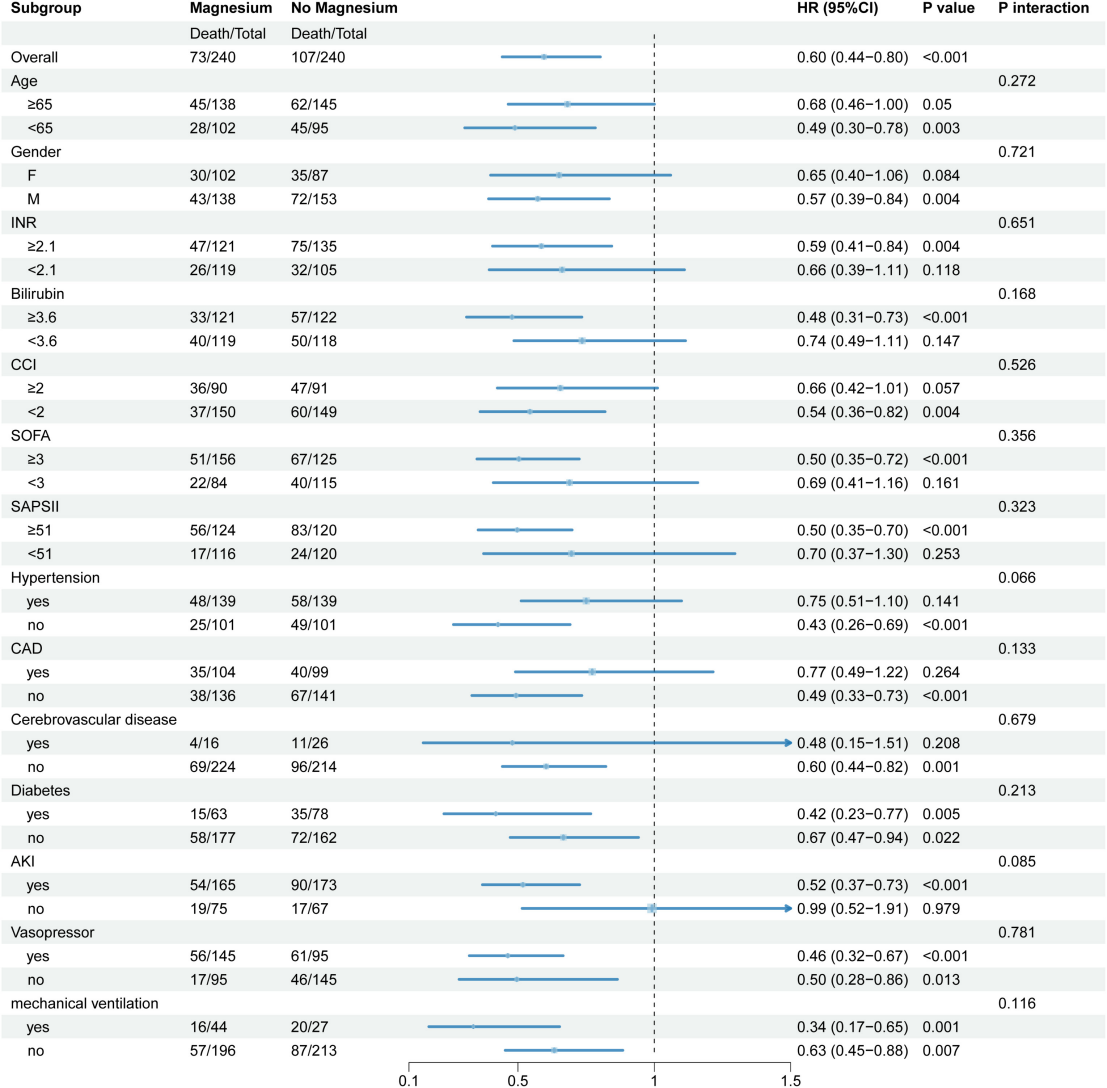
Subgroup analysis. Subgroup analysis used using a stratified Cox proportional hazards model, with the group not treated with magnesium sulfate as the common control group. For continuous variables, except for age, which was divided into two subgroups based on 65 years of age, all others were grouped based on the median. INR, international normalized ratio; CCI, charlson comorbidity index; SOFA, sequential organ failure assessment; SAPA II, Simplified Acute Physiology Score II; CAD, coronary artery disease; AKI, acute kidney injury; HR, hazard ratio; CI, confidence interval. *P*-value < 0.05 is considered statistically significant.

To further investigate the relationship between magnesium sulfate treatment and mortality in SALI patients with varying clinical profiles, a stratified Cox proportional hazards model was used for subgroup analyses, with the non-magnesium sulfate group serving as the reference. Continuous variables were categorized based on their median values, except for age, which was dichotomized at 65 years. Results indicate that the efficacy of magnesium sulfate was consistent across subgroups with differing characteristics (all interaction *P* > 0.05). Point estimates indicating a greater magnitude of effect were observed for magnesium therapy in reducing mortality risk among patients aged <65 years, males, those with INR > 2.1, total bilirubin ≥3.6 mg/dL, CCI < 2, SOFA > 3, SAPS II score ≥51, without hypertension, concomitant diabetes or AKI, and receiving vasopressors or mechanical ventilation, as shown in Figure 3.

**FIGURE 4:**
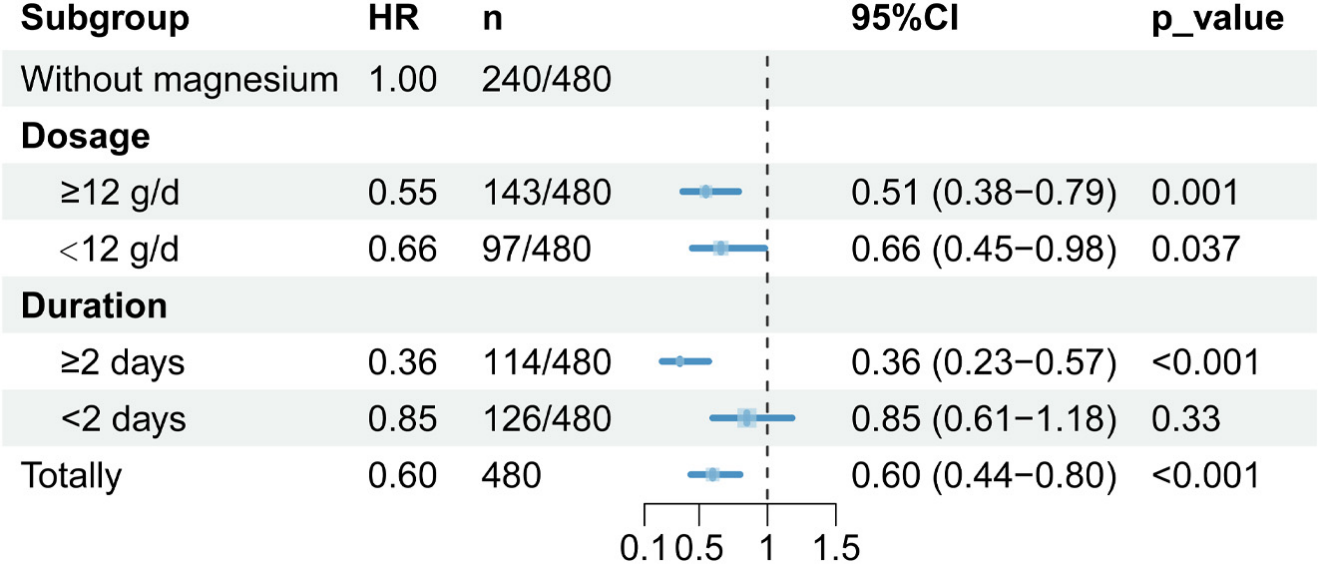
Subgroup analysis by the dosage and duration of magnesium sulfate. Subgroup analysis used a stratified Cox proportional hazards model, with dose and time grouped by median values, and 30-day mortality as the outcome. HR, hazard ratio; CI, confidence interval. *P*-value < 0.05 is considered statistically significant.

To evaluate the effects of different dosages and treatment durations of magnesium sulfate on mortality in SALI patients, we performed subgroup analyses with stratification by median of both dosage and duration, using 30-day mortality as the outcome. Results indicated that magnesium supplementation was associated with a 40% reduction in the risk of all-cause mortality (overall population HR = 0.60, 95% CI: 0.44–0.80, *p* < 0.001). Benefits were observed across the entire dose range studied, with a greater reduction in risk seen in the group receiving ≥12 g daily (HR = 0.55, *p* = 0.001); clinical benefit was observed in the group receiving treatment for ≥2 days (HR = 0.36, *p* < 0.001), as shown in Figure 4.

### 3.5 External validation results

The eicu2.0 database was utilized for external validation. A total of 241 patients with SALI (diagnostic criteria consistent with the previous description) were included, among whom 119 received magnesium sulfate treatment during their ICU stay. The baseline characteristics are presented in Supplementary Table 1. The results demonstrated that magnesium sulfate treatment significantly reduced in-hospital mortality, ICU mortality, and 30-day all-cause mortality. After adjusting for multiple confounding variables, the results from both IPW and Cox regression analyses remained consistent, as shown in Supplementary Table 2. This series of analyses indicates that the association between magnesium sulfate treatment and improved patient outcomes is robust.

## 4 Discussion

This study represents the first systematic assessment of the association between intravenous magnesium sulfate administration and outcomes in patients with SALI. Results revealed an overall 30-day mortality rate of 39.5% among SALI patients. After adjusting for confounding factors, magnesium sulfate therapy was significantly associated with reduced risks of 30-day and 90-day all-cause mortality, as well as ICU- and in-hospital mortality. This association remained consistent across different patient subgroups. These findings provide preliminary evidence for the potential therapeutic value of magnesium sulfate in SALI management.

Magnesium is an essential trace element that participates in vital physiological processes by regulating more than 300 enzyme-catalyzed reactions. Its deficiency has been proven to cause liver damage and various inflammatory reactions (19). Magnesium alleviates oxidative stress by inhibiting the production and accumulation of reactive oxygen species (ROS). Its deficiency exacerbates oxidative damage and inflammatory responses in the liver, leading to impaired liver cell function (20). Furthermore, hypomagnesemia is closely associated with abnormalities in glucose and lipid metabolism, promoting the accumulation of toxic substances in the liver (21). Magnesium deficiency is also accompanied by elevated levels of pro-inflammatory cytokines. The crucial role of magnesium in protein synthesis and its regulatory effects on innate immune responses further elucidate its multifaceted mechanisms in hepatic protection (22).

Magnesium deficiency represents a common comorbidity in liver diseases, and decreased magnesium levels in both serum and hepatic tissues can accelerate disease progression (23). Previous studies have confirmed that magnesium deficiency plays a significant role in the onset, progression, and outcome of various liver diseases, including metabolic dysfunction-associated fatty liver disease, alcoholic liver disease, liver fibrosis, and hepatocellular carcinoma. Magnesium supplementation, as a potential intervention, has demonstrated positive effects in clinical and animal studies by slowing disease progression and improving prognosis (24).

During the progression of sepsis, the liver undergoes a critical transition from an immunotolerant to an immunologically activated state, characterized by the substantial production of acute-phase proteins that induce systemic inflammatory response syndrome (SIRS) (6), a pathological condition closely associated with high mortality rates and intensive care requirements (25). Bacterial endotoxin (lipopolysaccharide, LPS) released from microbial membranes activates endoplasmic reticulum stress and triggers robust inflammatory cytokine responses (26), concurrently inducing hepatic lipid metabolism dysregulation and ROS overproduction, thereby exacerbating tissue damage and promoting hepatic failure (27). Previous animal studies have demonstrated that LPS administration induces systemic inflammation and hepatic dysfunction in murine models, manifested by elevated hepatic inflammatory cytokine levels and increased myeloperoxidase (MPO) activity, accompanied by enhanced lipid peroxidation, glutathione depletion, and elevated total nitrate levels along with heightened glutathione peroxidase activity. Notably, magnesium preconditioning has been shown to effectively attenuate sepsis-associated liver injury in these experimental models (28).

This study establishes the first evidence supporting the survival benefits of magnesium sulfate in SALI patients, though the exact mechanisms require further elucidation. The therapeutic effects may stem from pyroptosis inhibition and anti-inflammatory actions. Magnesium exerts protection through concurrent blockade of p38-MAPK, NF-κB, and Toll-like receptor 4 signaling pathways (29, 30), mitigating cytokine storms and inflammatory cascades. Additionally, it hydrolyzes membrane-bound CD14 to impede LPS-macrophage binding and inhibits ATP-gated P2 × 7 calcium channels (31), suppressing calcium efflux and gasdermin D N-terminal domain activation, thereby preventing LPS-induced atypical pyroptosis (9). These coordinated mechanisms collectively ameliorate hepatic histopathological damage and transaminase elevations, likely constituting the principal pathways through which magnesium alleviates liver injury.

In the field of critical care medicine, serum magnesium levels demonstrate significant correlations with clinical outcomes. Substantial evidence indicates that hypomagnesemic patients exhibit elevated mortality rates, increased requirements for mechanical ventilation, and prolonged hospital stays (32), whereas magnesium supplementation reduces mortality risk in this population (33). Particularly in septic patients, magnesium sulfate administration has been shown to markedly decrease both 28-day mortality and ICU mortality while reducing the need for renal replacement therapy (34). Additionally, magnesium therapy exhibits cardioversion efficacy in patients with new-onset atrial fibrillation and demonstrates neuroprotective properties (35, 36), warranting its broad clinical recommendation in the management of stroke and traumatic brain injury patients.

Overall subgroup analysis results suggest that the beneficial effects of magnesium therapy may be more pronounced in certain specific populations, such as younger patients, those with higher disease severity (e.g., SOFA ≥3, SAPS II ≥51, use of vasopressors or mechanical ventilation), and those without a history of hypertension. However, the study sample size was not designed for these subgroup analyses, with many subgroups being too small to achieve sufficient statistical power to detect genuine interactions. We therefore refrain from asserting that magnesium is more effective in any specific subgroup, instead viewing these findings as signals generating hypotheses to guide future study designs targeting particular populations. Furthermore, subgroup analyses by time and dose suggested that adequate treatment duration (≥ 2 days) is crucial for achieving clinical benefit from magnesium supplementation, while higher doses (≥ 12 g/day) may yield superior therapeutic effects. Therefore, in clinical practice, the dosage and duration of magnesium treatment should be adjusted according to the specific conditions of patients to maximize therapeutic efficacy. These findings provide empirical support for the clinical application of magnesium, highlighting the need for future research to elucidate the underlying mechanisms and evaluate the long-term effects of magnesium intervention.

Furthermore, we employed RCS modeling to examine the associations between INR and TB levels and 30-day mortality, revealing significant correlations for both parameters. The RCS curve for INR demonstrated a predominantly linear ascending trend, indicating progressively elevated mortality risk with increasing INR values, particularly when exceeding 2.0, suggesting coagulopathy substantially contributes to adverse outcomes. In contrast, the bilirubin RCS curve exhibited a characteristic U-shaped non-linear relationship, with higher mortality risks observed at both lower (approximately 2–3 mg/dL) and elevated (> 5 mg/dL) concentrations. Markedly increased bilirubin levels reflect either severe hepatic dysfunction or biliary obstruction, potentially signifying disease progression. These findings underscore the clinical importance of closely monitoring both parameters and implementing early interventions for high-risk patients.

In summary, magnesium has emerged as a multifunctional trace element with increasingly recognized hepatoprotective effects in liver diseases, particularly critical illness-associated hepatic injury, mediated through its pleiotropic mechanisms including metabolic regulation, antioxidant activity, anti-inflammatory actions, and hepatocyte homeostasis maintenance. Future clinical investigations should aim to establish standardized indications, optimal dosing regimens, and validated efficacy endpoints for magnesium supplementation, thereby facilitating its integration as a novel therapeutic strategy in the comprehensive management of hepatic disorders.

This study holds significant clinical relevance by highlighting the necessity for enhanced surveillance of serum magnesium concentrations in critically ill patients, especially those diagnosed with sepsis; it provides preliminary evidence for the value of magnesium sulfate as an adjunctive therapy for SALI, with high-dose, long-course regimens may offer greater advantages. These results provide practical guidance for improving SALI patient outcomes, especially in resource-constrained clinical settings. Given its low cost and favorable safety profile, the judicious use of magnesium sulfate may serve as an effective therapeutic strategy to enhance treatment success rates.

This study has several limitations: firstly, despite using methods such as propensity score matching and multivariate regression analysis, residual bias and unmeasured confounding variables may persist in the results, as factors including diuretic use, chronic kidney disease, and renal replacement therapy could influence magnesium metabolism and patient outcomes; second, this is a retrospective study, which can only demonstrate the association between magnesium sulfate therapy and mortality in SALI patients, but cannot establish a causal relationship; third, the MIMIC-IV database does not record information regarding the indications for magnesium sulphate use, discontinuation criteria, or the administration of magnesium supplements before hospital admission; Fourth, individual differences and dynamic changes in patient conditions were not assessed, which may have influenced the results; finally, the data in this study were sourced from a single centre in the United States and require validation through external cohorts to assess their scientific validity across different populations and regions. Future research should validate the results through large-scale, prospective, multicenter studies to clarify the therapeutic effects and clinical benefits of magnesium sulfate in SALI patients.

## 5 Conclusion

Magnesium sulfate supplementation therapy was significantly associated with a reduction in mortality risk among SALI patients. This finding suggests the potential of magnesium sulfate as an adjunctive treatment for SALI; however, its precise efficacy and individualized application strategies require further validation through prospective randomized controlled trials.

## Data Availability

Publicly available datasets were analyzed in this study. This data can be found here: https://physionet.org/content/mimiciv/3.1/.
