# Supplementary material for: Association between intravenous magnesium sulfate and mortality in patients with sepsis-associated liver injury: a retrospective cohort study
Source: Front Med (Lausanne). 2026 Jan 13;12:1679032. doi: 10.3389/fmed.2025.1679032 (PMC12835258; doi:10.3389/fmed.2025.1679032)
Supplement: Supplementary file 1 [file Data_Sheet_1.docx]

Supplementary Material


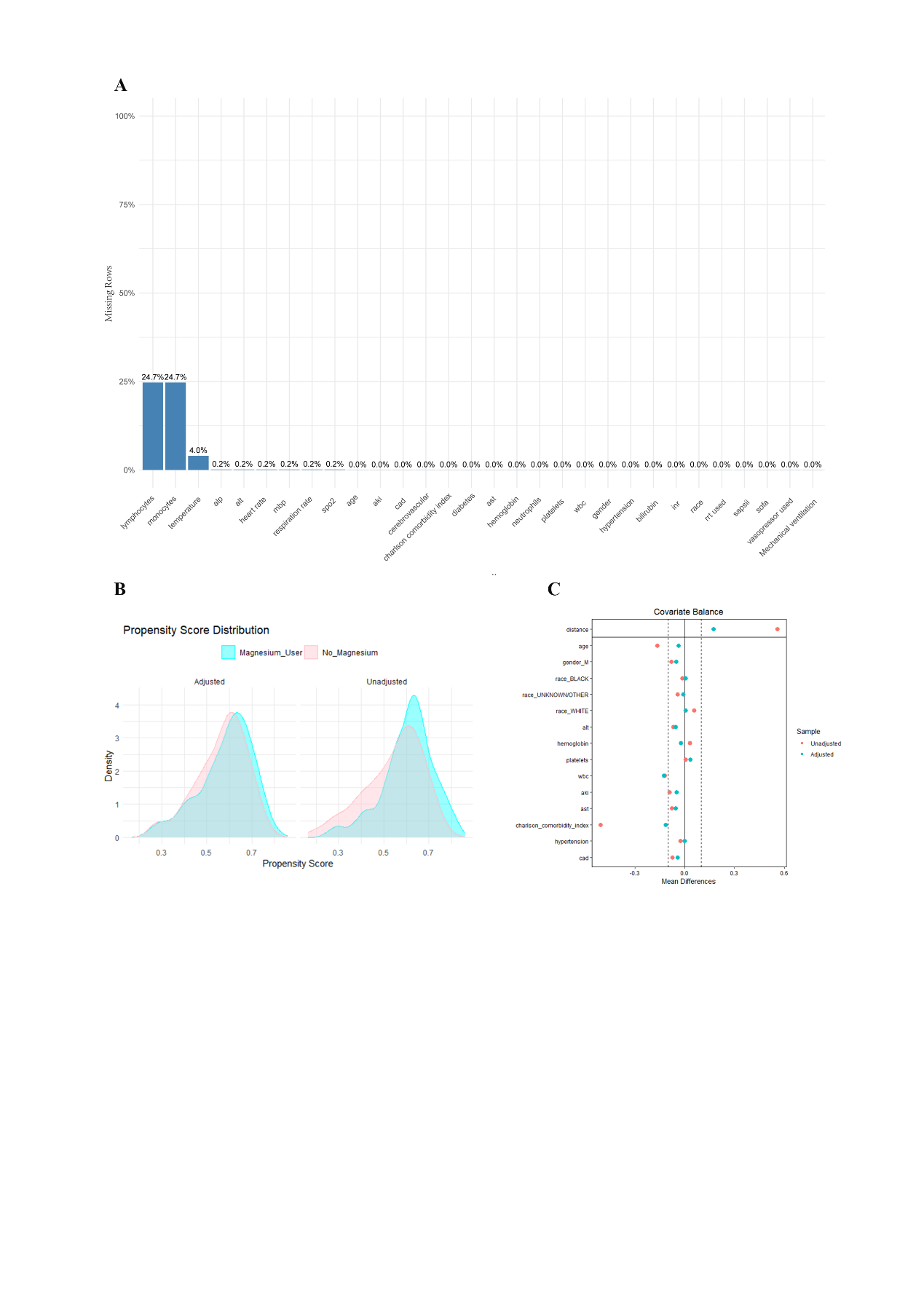


**Supplementary Figure S1:Data Preparation.** Figure A shows the Missing value percentage per variable; variables with >25% missing data were excluded. Figure B shows preference score distribution. A higher degree of overlap indicates that patients in the target group and control group are more similar in terms of the likelihood of receiving the target treatment. Figure C. shows the distribution of each covariate before and after PSM. The absolute mean differences were used to assess the balance of confounding variables, with a threshold of >10% set as the criterion for significant imbalance. ALT: Alanine Aminotransferase; ALP: Alkaline Phosphatase; MBP: Mean Blood Pressure; SpO_2_: Oxygen Saturation; AKI: Acute Kidney Injury; CAD: Coronary Artery Disease; AST: Aspartate Aminotransferase; WBC: White Blood Cell; INR: International Normalized Ratio; RRT: Renal Replacement Therapy; SAPS II: Simplified Acute Physiology Score II; SOFA: Sequential Organ Failure Assessment; PSM: Propensity Score Matching.


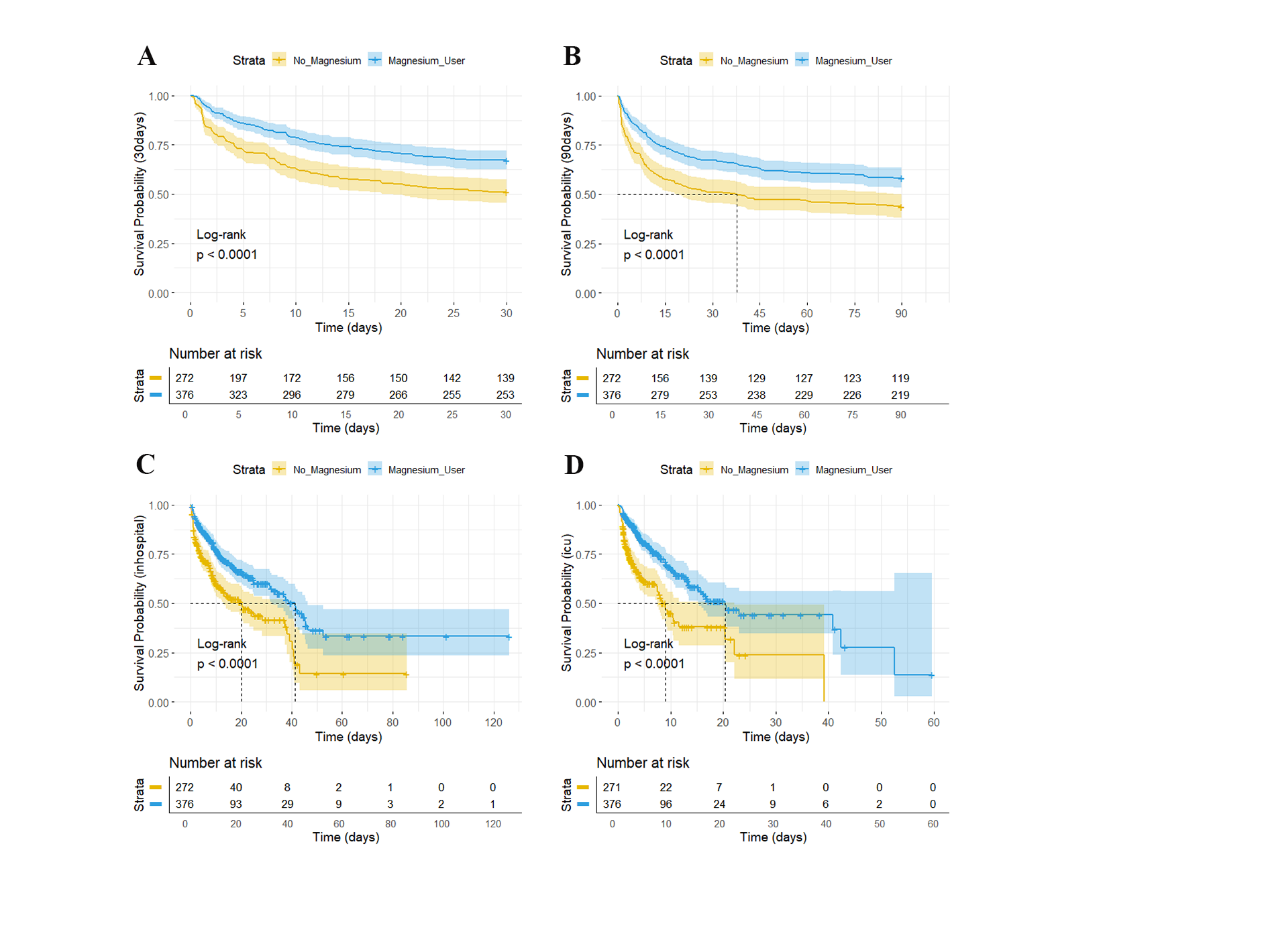


**Supplementary Figure S2: Kaplan-Meier Survival Analysis before PSM.** Kaplan–Meier curves (log-rank test) show 30-day survival probability (A), 90-day survival probability (B), in-hospital survival probability (C), and ICU survival probability (D), grouped by Magnesium sulfate used. The X-axis denotes the time (days), and the Y-axis denotes the cumulative survival probability. ICU: Intensive Care Unit; PSM: Propensity Score Matching. P value < 0.05 is considered statistically significant.


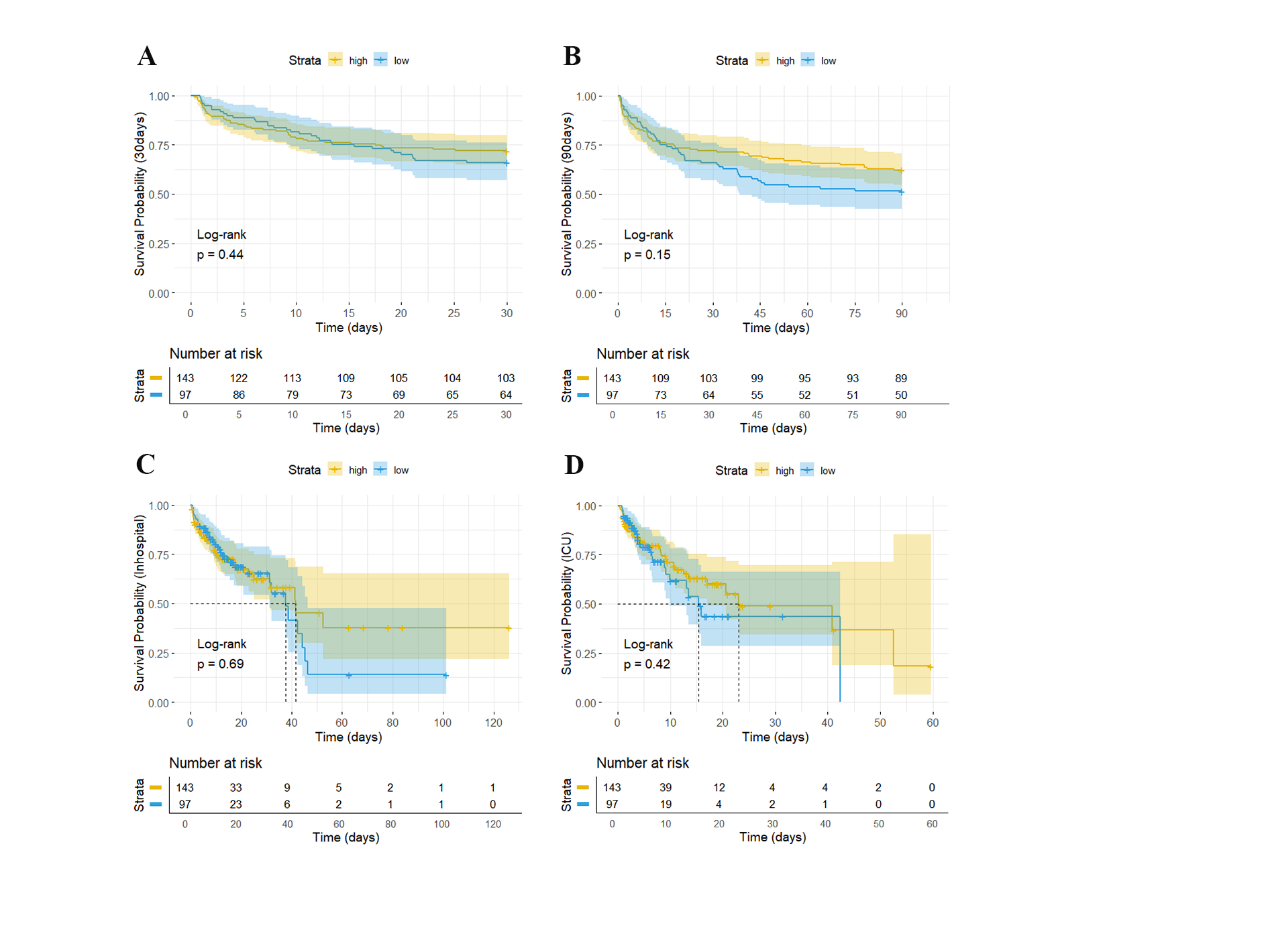


**Supplementary Figure S3:** **Kaplan-Meier Survival Analysis by Dosage of Magnesium Sulfate.** Kaplan–Meier curves (log-rank test) show 30-day survival probability (A), 90-day survival probability (B), in-hospital survival probability (C), and ICU survival probability (D), grouped by Magnesium sulfate dosage. The X-axis denotes the time (days), and the Y-axis denotes the cumulative survival probability. ICU: Intensive Care Unit. P value < 0.05 is considered statistically significant.


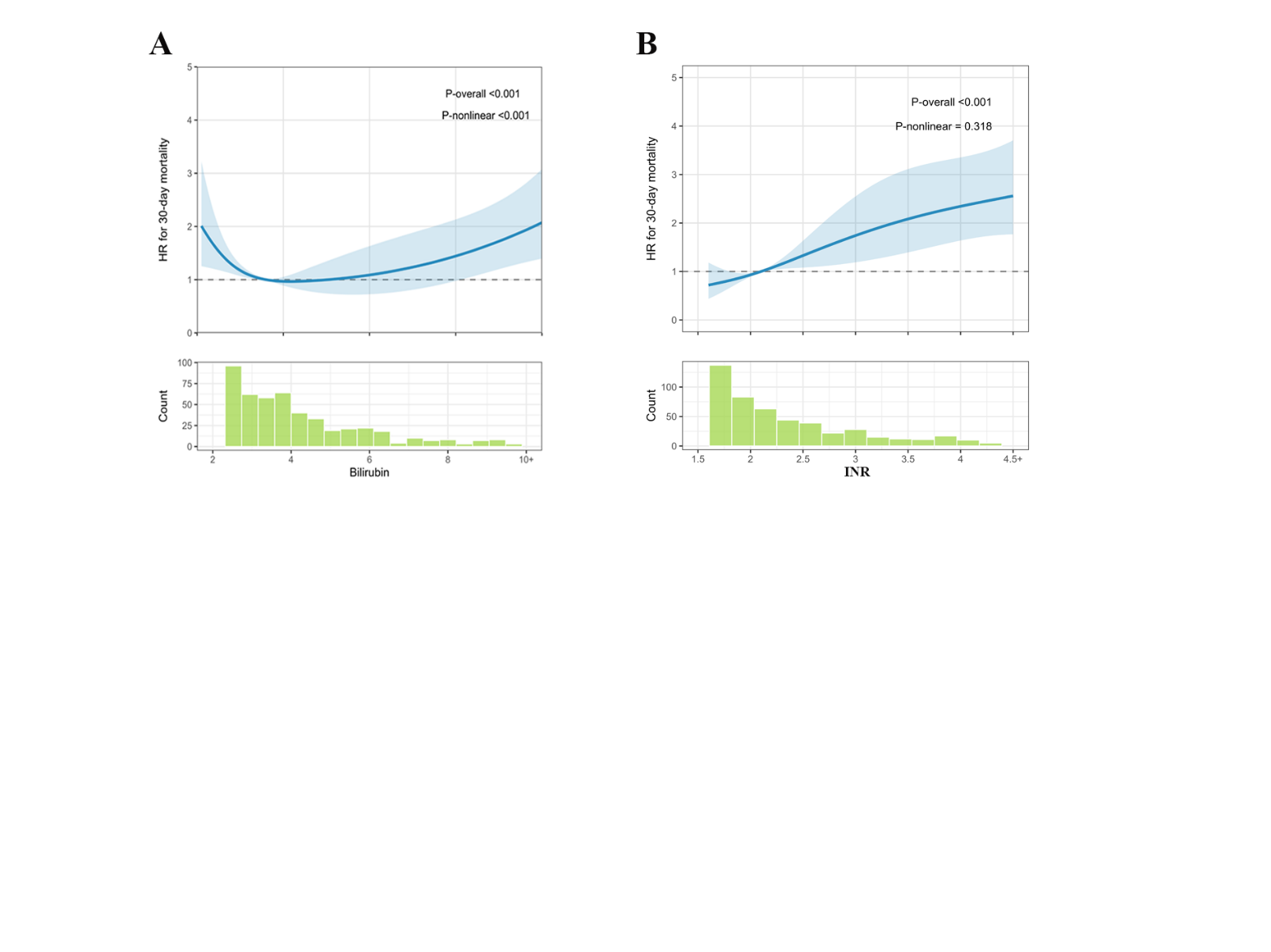


**Supplementary Figure S4: Association Between INR or Total Bilirubin and 30-day Mortality in Patients with SALI.** Graphs show HRs between total bilirubin (A) or INR (B) and 30 days mortality. Data were fitted by a restricted cubic spline Cox proportional hazards regression model. Solid lines indicate HRs, and shadow shapes indicate 95% Cis. INR: International Normalized Ratio; HR: hazard ratio; CI: confidence interval. P value <0.05 is considered statistically significant.

| Variables | No magnesium | Magnesium | p |
| --- | --- | --- | --- |
| Patients number | 122 | 119 |  |
| **Demographics** |  |  |  |
| age | 61.50 (52.00, 71.75) | 61.00 (49.00, 69.00) | 0.513 |
| gender，Male | 74 (60.7) | 67 (56.3) | 0.515 |
| Race |  |  | 0.496 |
| Black | 11 ( 9.0) | 10 ( 8.4) |  |
| white | 95 (77.9) | 99 (83.2) |  |
| Other/Unknown | 16 (13.1) | 10 ( 8.4) |  |
| **Laboratory variables** |  |  |  |
| Heart rate | 92.18 (81.20, 102.31) | 91.97 (84.97, 102.88) | 0.465 |
| SBP | 111.75 (104.34, 123.01) | 112.27 (103.09, 122.69) | 0.812 |
| DBP | 58.00 (50.58, 63.57) | 56.30 (50.91, 63.53) | 0.835 |
| Respiratory | 21.56 (17.86, 24.28) | 21.21 (18.45, 24.00) | 0.874 |
| Temperature | 36.40 (35.82, 36.72) | 36.40 (36.00, 36.75) | 0.895 |
| SpO2 | 96.75 (94.95, 97.86) | 97.20 (95.89, 98.13) | 0.078 |
| WBC | 16.35 (8.75, 24.62) | 13.49 (7.89, 22.33) | 0.129 |
| Platelets | 114.50 (65.50, 205.75) | 126.00 (58.50, 190.50) | 0.957 |
| ALT | 49.00 (28.00, 143.50) | 54.00 (32.50, 178.50) | 0.255 |
| AST | 81.00 (42.00, 207.00) | 119.00 (54.00, 328.00) | 0.074 |
| INR | 2.56 (2.00, 3.27) | 2.47 (1.80, 3.80) | 0.983 |
| Bilirubin | 5.25 (3.42, 11.47) | 6.10 (3.20, 10.40) | 0.724 |
| CCI | 4.00(2.00,6.00) | 4.00(1.00,5.00) | 0.192 |
| **Comorbidity** |  |  |  |
| Hypertension | 67 (54.9) | 54 (45.4) | 0.157 |
| CAD | 6 ( 4.9) | 10 ( 8.4) | 0.311 |
| Cerebrovascular | 12 ( 9.8) | 4 ( 3.4) | 0.068 |
| Diabetes | 32 (26.2) | 30 (25.2) | 0.884 |
| AKI | 57 (46.7) | 57 (47.9) | 0.898 |
| **Critical treatments** |  |  |  |
| vasopressor | 56 (45.9) | 75 (63.0) | 0.010 |
| RRT | 27 (22.1) | 30 (25.2) | 0.650 |
| Mechanical ventilator | 19 (15.6) | 7 ( 5.9) | 0.021 |

**Table S1:** **The baseline characteristic of eICU database.** SBP: Systolic Blood Pressure; DBP: Diastolic Blood Pressure; SpO_2_: Oxygen Saturation; WBC: White Blood Cell; ALT: Alanine Aminotransferase; AST: Aspartate Aminotransferase; INR: International Normalized Ratio; CCI: Charlson Comorbidity Index; CAD: Coronary Artery Disease; CVD: cerebrovascular disease; AKI: Acute Kidney Injury; RRT: Renal Replacement Therapy. P value <0.05 is considered statistically significant.

| mortality | MODEL | HR(%95cl) | P value |
| --- | --- | --- | --- |
| 30-day mortality | COX | 0.62 (0.38-0.99) | 0.043 |
|  | IPW | 0.62 (0.40-0.98) | 0.039 |
| in hospital mortality | COX | 0.60 (0.38-0.95) | 0.03 |
|  | IPW | 0.63 (0.41-0.97) | 0.035 |
| ICU mortality | COX | 0.45 (0.25-0.79) | 0.005 |
|  | IPW | 0.46 (0.27-0.78) | 0.004 |

**Table S2: Association of magnesium sulfate with all-cause mortality of SALI patients in eICU database.** Conducted Cox proportional hazards regression and IPW analysis using the outcomes of death during hospitalization, death within 30 days, and death during ICU stay. The model was adjusted by age, gender, respiratory Rate, systolic blood pressure, heart rate, white blood cell, total bilirubin, cerebrovascular disease, coronary artery disease, diabetes, vasopressor used, renal replacement therapy and CCI score.
